# Correction: Association between long COVID and nonsteroidal anti-inflammatory drug use by patients with acute-phase COVID-19: A nationwide Korea National Health Insurance Service cohort study

**DOI:** 10.1371/journal.pone.0328398

**Published:** 2025-07-15

**Authors:** The PLOS One Staff

The following information is missing from the Data Availability statement: This study used NHIS-NSC data (NHIS-2023-1-375) made by National Health Insurance Service (NHIS). The authors declare no conflict of interest with NHIS.

The publisher apologizes for the error.

## References

[1] Lee Y-S, Kim H, Kwon S, Kim T-H. Association between long COVID and nonsteroidal anti-inflammatory drug use by patients with acute-phase COVID-19: A nationwide Korea National Health Insurance Service cohort study. PLoS One. 2024;19(11):e0312530. doi: 10.1371/journal.pone.0312530 39576781 PMC11584118

