# Supplementary material for: Association between long COVID and nonsteroidal anti-inflammatory drug use by patients with acute-phase COVID-19: A nationwide Korea National Health Insurance Service cohort study
Source: PLoS One. 2024 Nov 22;19(11):e0312530. doi: 10.1371/journal.pone.0312530 (PMC11584118; doi:10.1371/journal.pone.0312530)
Supplement: S1 Table — AIDS, acute immunodeficiency syndrome; COVID-19, coronavirus disease-2019; HIV, human immunodeficiency virus; NSAIDs, nonsteroidal anti-inflammatory drugs. (DOCX) [file pone.0312530.s001.docx]

**Supplementary table 1. Study information**

| **Diagnosis** | **ICD-10-CM code or ATC code & definition** | |
| --- | --- | --- |
| **Inclusion criteria** |  |  |
| COVID-19 | U07.1, U07.2, B34.2, B97.2 | Initial diagnosis in 03/01/2020 - 12/31/2021 [entry date] |
| **Exclusion criteria** |  |  |
| Death |  | Reports of death within entry date + 90 |
| **Exposures** |  |  |
| NSAIDs | M01A, B01A | Acute period since COVID [entry date, entry date + 15] |
| Acetaminophen | N02A, N02B |  |
| Antiviral drugs | J05A |  |
| **Baseline covariates** |  | |
| **Comorbid conditions within one year before the index date** | | |
| Diabetes mellitus (both with and without complications) | E10, E11, E12, E13, E14 | [Entry date-90, Entry date] |
| Osteoarthritis | M15, M16, M17, M18, M19 |  |
| Rheumatoid | M05, M06 |  |
| Osteoporosis | M80, M81, M82 |  |
| Chronic obstructive pulmonary disease | J43, J44 |  |
| Dementia | F00, F01, F02, F03, G30 |  |
| Depressive disorders | F32, F33 |  |
| Anxiety disorders | F40, F41 |  |
| Sleep disorder | G47, F51 |  |
| Hyperlipidemia | E78 |  |
| Hypertension | I10, I11, I12, I13, I15 |  |
| Other cardiovascular diseases | I05, I06, I07, I08, I09, I23, I24, I25, I26, I27, I30, I31, I32, I33, I34, I35, I36, I37, I38, I39, I40, I41, I43, I44, I45, I47, I48, I49, I51, I52 |  |
| Renal failure | N17, N18, N19 |  |
| Chronic liver diseases | K72, K73, K74, K75, K76, K77 |  |
| Cerebrovascular disease | I60, I61, I62, I63, I64, I65, I66, I67, I68, I69 |  |
| Anemia | D50, D51, D52, D53, D55, D56, D57, D58, D59, D60, D61, D62, D63, D64 |  |
| **Charlson Comorbidity Index (Score)** |  | |
| Myocardial infarction | I21, I22, I252 | [Entry date-90, Entry date] |
| Congestive heart failure | I099, I110, I130, I132, I255, I420, I425, I426, I427, I428, I429, I43, I50, P290 |  |
| Peripheral vascular disease | I70, I71, I731, I738, I739, I771, I790, I792, K551, K558, K559, Z958, Z959 |  |
| Cerebrovascular disease | G45, G46, I60, I61, I62, I63, I64, I65, I66, I67, I68, I69, H340 |  |
| Dementia | F00, F01, F02, F03, G30, F051, G311 |  |
| Chronic pulmonary disease | I278, I279, J40, J41, J42, J43, J44, J45, J46, J47, J60, J61, J62, J63, J64, J65, J66, J67, J684, J701, J703 |  |
| Connective tissue disease | M05, M06, M315, M32, M33, M34, M351, M353, M360 |  |
| (Rheumatologic disease) |  |  |
| Peptic ulcer disease | K25, K26, K27, K28 |  |
| Mild liver disease | B18, K700, K701, K702, K703, K709, K713, K714, K715, K717, K73, K74, K760, K762, K763, K764, K768, K769, Z944 |  |
| Moderate or severe liver disease (3) | I850, I859, I864, I982, K704, K711, K721, K729, K765, K766, K767 |  |
| Diabetes without complications | E100, E101, E106, E108, E109, E110, E111, E116, E118, E119, E120, E121, E126, E128, E129, E130, E131, E136, E138, E139, E140, E141, E146, E148, E149 |  |
| Diabetes with complications (2) | E102, E103, E104, E105, E107, E112, E113, E114, E115, E117, E122, E123, E124, E125, E127, E132, E133, E134, E135, E137, E142, E143, E144, E145, E147 |  |
| Paraplegia and hemiplegia (2) | G041, G114, G800, G801,G802, G81, G82, G830, G831, G832, G833, G834, G839 |  |
| Renal disease (2) | I120, I131, N030, N031, N032, N033, N034, N035, N036, N037, N038, N039, N050, N051, N052, N053, N054, N055, N056, N057, N058, N059, N18, N19, N250, Z490, Z491, Z492, Z940, Z992 |  |
| Cancer (2) | C00, C01, C02, C03, C04, C05, C06, C07, C08, C09, C10, C11, C12, C13, C14, C15, C16, C17, C18, C19, C20, C21, C22, C23, C24, C25, C26, C30, C31, C32, C33, C34, C37, C38, C39, C40, C41, C43, C45, C46, C47, C48, C49, C50, C51, C52, C53, C54, C55, C56, C57, C58, C60, C61, C62, C63, C64, C65, C66, C67, C68, C69, C70, C71, C72, C73, C74, C75, C76, C81, C82, C83, C84, C85, C88, C90, C91, C92, C93, C94, C95, C96, C97 |  |
| Metastatic carcinoma (6) | C77, C78, C79, C80 |  |
| AIDS/HIV (6) | B20, B21, B22, B24 |  |
| **Demographic variables** |  |  |
| Age group | <45, 45-54, 55-64, ≥65 | [Entry date] |
| Area | Seoul |  |
|  | Metropolitan City |  |
|  | Province |  |
|  | Quarantine station |  |
| **Outcome of interest** |  |  |
| **Long COVID** | U09 | [Entry date+90, Dec 31 2021] |

COVID, coronavirus disease-2019; NSAIDs, non-steroidal anti-inflammatory drugs; AIDS, acute immunodeficiency syndrome; HIV, human immunodeficiency virus.
