# Supplementary material for: Association between long COVID and nonsteroidal anti-inflammatory drug use by patients with acute-phase COVID-19: A nationwide Korea National Health Insurance Service cohort study
Source: PLoS One. 2024 Nov 22;19(11):e0312530. doi: 10.1371/journal.pone.0312530 (PMC11584118; doi:10.1371/journal.pone.0312530)
Supplement: S2 Table — Model 1: univariate; Model 2: adjusted for COVID duration; Model 3: adjusted for duration, sex, and age group; Model 4: adjusted for COVID duration, sex, age group, and region; Model 5: adjusted for duration, sex, age group, region, and CCI; Model 6: adjusted for COVID duration, sex, age group, region, CCI, and underlying comorbidities. 1Day of COVID diagnosis to 14 days later. 215 days since COVID diagnosis to end of follow-up (diagnosis of long COVID, diagnosis of other COVID episodes, or end of observation period). *p < 0.05; **p < 0.01; ***p < 0.001. APAP, acetaminophen or paracetamol; CCI, Charlson Comorbidity Index; CI, confidence interval; NSAIDs, nonsteroidal anti-inflammatory drugs; OR, odds ratio. (DOCX) [file pone.0312530.s002.docx]

**Supplementary Table 2. Univariate and multivariate logistic models of medication exposure between long COVID cases and controls with duration of COVID as a covariate**

|  | **After Propensity Score Matching** | | | | | |
| --- | --- | --- | --- | --- | --- | --- |
|  | **Model 1 (Unadjusted)** | **Model 2** | **Model 3** | **Model 4** | **Model 5** | **Model 6** |
|  | **OR (95% CI)** | **OR (95% CI)** | **OR (95% CI)** | **OR (95% CI)** | **OR (95% CI)** | **OR (95% CI)** |
| **NSAIDs (ref. non-users)** | | | | | | |
| Acute exposure^1^ | 1.759 (0.996-3.107) | 1.711 (0.965-3.034) | 1.716 (0.964-3.054) | 1.728 (0.969-3.082) | 1.739 (0.973-3.108) | 1.636 (0.895-2.990) |
| Chronic exposure^2^ | 0.598 (0.439-0.816) ** | 0.601 (0.441-0.820) ** | 0.591 (0.431-0.810) ** | 0.591 (0.431-0.811) ** | 0.589 (0.429-0.809) ** | 0.541 (0.385-0.759) *** |
| **APAP (ref. non-users)** | | | | | | |
| Acute exposure^1^ | 1.128 (0.774-1.643) | 1.112 (0.763-1.622) | 1.108 (0.756-1.624) | 1.115 (0.755-1.647) | 1.118 (0.753-1.659) | 1.100 (0.733-1.650) |
| Chronic exposure^2^ | 0.658 (0.464-0.932) * | 0.660 (0.465-0.935) * | 0.653 (0.459-0.929) * | 0.653 (0.459-0.929) * | 0.652 (0.458-0.928) * | 0.595 (0.410-0.862) ** |
| **Antiviral drugs(ref. non-users)** | | | | | | |
| Acute exposure^1^ | 3.582 (1.681-7.636) ** | 3.488 (1.631-7.459) ** | 3.588 (1.654-7.786) ** | 3.618 (1.665-7.864) ** | 3.630 (1.667-7.903) ** | 3.629 (1.597-8.248) ** |
| Chronic exposure^2^ | - | - | - | - | - |  |

Model 1, univariate; Model 2, adjusted for COVID duration; Model 3, adjusted for COVID duration, sex, and age group; Model 4, adjusted for COVID duration, sex, age group, and region; Model 5, adjusted for duration, sex, age group, region, and CCI; Model 6, adjusted for COVID duration, sex, age group, region, CCI, and underlying comorbidities

^1^Day of COVID diagnosis - 14 days after COVID diagnosis

^2^15 days since COVID diagnosis - end of follow-up (diagnosis of long COVID, diagnosis of other COVID episodes, or end of observation period

**p* <0.05; ***p* <0.01; ****p* <0.001

NSAIDs, nonsteroidal anti-inflammatory drugs; CI, confidence interval; OR, odds ratio; APAP, acetaminophen or paracetamol
