# Supplementary material for: Association between long COVID and nonsteroidal anti-inflammatory drug use by patients with acute-phase COVID-19: A nationwide Korea National Health Insurance Service cohort study
Source: PLoS One. 2024 Nov 22;19(11):e0312530. doi: 10.1371/journal.pone.0312530 (PMC11584118; doi:10.1371/journal.pone.0312530)
Supplement: S3 Table — Model 1: univariate; Model 2: adjusted for sex and age group; Model 3: adjusted for sex, age group, and region; Model 4: adjusted for sex, age group, region, and CCI; and Model 5: adjusted for sex, age group, region, CCI, and underlying comorbidities. 1Day of COVID diagnosis to 14 days later. 215 days since COVID diagnosis to end of follow-up (diagnosis of long COVID, diagnosis of other COVID episodes, or end of observation period). *p < 0.05; **p < 0.01; ***p < 0.001. APAP, acetaminophen or paracetamol; CI, confidence interval; NSAIDs, nonsteroidal anti-inflammatory drugs; OR, odds ratio. (DOCX) [file pone.0312530.s003.docx]

**Supplementary Table 3. Univariate and multivariate logistic models comparing single NSAID users with acetaminophen or paracetamol (APAP) users and combined nonsteroidal anti-inflammatory drugs + APAP with APAP users**

|  | **After Propensity Score Matching** | | | | |
| --- | --- | --- | --- | --- | --- |
|  | **Model 1 (Unadjusted)** | **Model 2** | **Model 3** | **Model 4** | **Model 5** |
|  | **OR (95% CI)** | **OR (95% CI)** | **OR (95% CI)** | **OR (95% CI)** | **OR (95% CI)** |
| **NSAIDs single users (ref. Acetaminophen single users)** | | | | | |
| Acute exposure^1^ | 1.858 (0.625-5.527) | 1.828 (0.593-5.637) | 2.017 (0.622-6.541) | 2.503 (0.706-8.876) | 2.805 (0.616-12.771) |
| Chronic exposure^2^ | 0.939 (0.528-1.668) | 0.976 (0.540-1.765) | 0.962 (0.529-1.748) | 0.932 (0.509-1.705) | 1.017 (0.529-1.956) |
| **Single or combined NSAIDs + APAP users (ref. APAP single users)** | | | | | |
| Acute exposure^1^ | 1.770 (0.894-3.502) | 1.725 (0.856-3.477) | 1.871 (0.881-3.975) | 1.889 (0.877-4.067) | 1.924 (0.838-4.418) |
| Chronic exposure^2^ | 0.924 (0.541-1.578) | 0.945 (0.549-1.627) | 0.933 (0.541-1.609) | 0.921 (0.533-1.593) | 0.998 (0.558-1.784) |

Model 1: univariate; Model 2: adjusted for sex and age group; Model 3: adjusted for sex, age group, and region; Model 4: adjusted for sex, age group, region, and CCI; Model 5: adjusted for sex, age group, region, CCI, and underlying comorbidities

^1^Day of COVID diagnosis - 14 days after COVID diagnosis

^2^15 days since COVID diagnosis - end of follow-up (diagnosis of long COVID, diagnosis of other COVID episodes, or end of observation period

**p* <0.05; ***p* <0.01; ****p* <0.001

NSAIDs, nonsteroidal anti-inflammatory drugs; CI, confidence interval; OR, odds ratio; APAP, acetaminophen or paracetamol
