# Supplementary material for: Association between long COVID and nonsteroidal anti-inflammatory drug use by patients with acute-phase COVID-19: A nationwide Korea National Health Insurance Service cohort study
Source: PLoS One. 2024 Nov 22;19(11):e0312530. doi: 10.1371/journal.pone.0312530 (PMC11584118; doi:10.1371/journal.pone.0312530)
Supplement: Attachment — Submitted filename: Comments_PONE-D-24-33236.docx [file pone.0312530.s005.docx]

**Reviewer Comments**

Association between long COVID and acute usage of nonsteroidal anti-inflammatory 2 drugs (NSAIDs) in COVID-19 patients: a nationwide cohort study from the National 3 Health Insurance Service in Korea

**Concerns**

1. The manuscript will benefit from a grammar check.
2. Rephrase your conclusion in the abstract. Should be straightforward….eg., Both NSAIDS and APAP are robustly associated with reduced risk of Long-term COVID-19…..
3. How and where were the controls recruited? Why did the controls have more medical conditions (covariates) than the cases? Could this have affected the outcomes observed in model 5 of Table 2? Could this potential protective effect be due to chance?
4. Your recommendations should be more specific and not generalized. Which specific molecular or biochemical aspects are required to expand on observed relationships?

**Decision: Minor revision**
