# Supplementary material for: Association between long COVID and nonsteroidal anti-inflammatory drug use by patients with acute-phase COVID-19: A nationwide Korea National Health Insurance Service cohort study
Source: PLoS One. 2024 Nov 22;19(11):e0312530. doi: 10.1371/journal.pone.0312530 (PMC11584118; doi:10.1371/journal.pone.0312530)
Supplement: Attachment — Submitted filename: renamed_72265.docx [file pone.0312530.s006.docx]

**Answer to the editor and reviewers’ comments**

**Editor’s comment**

- **A rebuttal letter that responds to each point raised by the academic editor and reviewer(s). You should upload this letter as a separate file labeled 'Response to Reviewers'.**

Thank you for your comments. We did this.

- **A marked-up copy of your manuscript that highlights changes made to the original version. You should upload this as a separate file labeled 'Revised Manuscript with Track Changes'.**

Thank you for your comments. We did this.

- **An unmarked version of your revised paper without tracked changes. You should upload this as a separate file labeled 'Manuscript'.**

Thank you for your comments. We did this.

**If you would like to make changes to your financial disclosure, please include your updated statement in your cover letter. Guidelines for resubmitting your figure files are available below the reviewer comments at the end of this letter.**

We didn’t change to our financial disclosure.

**If applicable, we recommend that you deposit your laboratory protocols in** [**protocols.io**](http://protocols.io) **to enhance the reproducibility of your results.** [**Protocols.io**](http://Protocols.io) **assigns your protocol its own identifier (DOI) so that it can be cited independently in the future. For instructions see:** [**https://journals.plos.org/plosone/s/submission-guidelines#loc-laboratory-protocols**](https://c05y1x9s.r.us-east-2.awstrack.me/L0/https:%2F%2Fjournals.plos.org%2Fplosone%2Fs%2Fsubmission-guidelines%23loc-laboratory-protocols/1/010f01918f98a0e5-8bf41aab-3a91-43c4-8259-5f0df08c0615-000000/MK0cK930bTONYDUWW0tvocVpYhk=173)**. Additionally, PLOS ONE offers an option for publishing peer-reviewed Lab Protocol articles, which describe protocols hosted on** [**protocols.io**](http://protocols.io)**. Read more information on sharing protocols at** [**https://plos.org/protocols?utm_medium=editorial-email&utm_source=authorletters&utm_campaign=protocols**](https://c05y1x9s.r.us-east-2.awstrack.me/L0/https:%2F%2Fplos.org%2Fprotocols%3Futm_medium=editorial-email%26utm_source=authorletters%26utm_campaign=protocols/1/010f01918f98a0e5-8bf41aab-3a91-43c4-8259-5f0df08c0615-000000/aSFPqdO09cGjXI_26LayZv4KMqw=173)**.**

Thank you for your comments.

1. **Please ensure that your manuscript meets PLOS ONE's style requirements, including those for file naming. The PLOS ONE style templates can be found at**
   [**https://journals.plos.org/plosone/s/file?id=wjVg/PLOSOne_formatting_sample_main_body.pdf**](https://journals.plos.org/plosone/s/file?id=wjVg/PLOSOne_formatting_sample_main_body.pdf) **and** 
   [**https://journals.plos.org/plosone/s/file?id=ba62/PLOSOne_formatting_sample_title_authors_affiliations.pdf**](https://journals.plos.org/plosone/s/file?id=ba62/PLOSOne_formatting_sample_title_authors_affiliations.pdf)

We followed the Plos one’s recommendation for formatting.

**2. Thank you for stating the following in the Acknowledgments Section of your manuscript: 
   "This study was supported by the Korea Institute of Oriental Medicine [grant number KSN2121220]."
We note that you have provided funding information that is not currently declared in your Funding Statement. However, funding information should not appear in the Acknowledgments section or other areas of your manuscript. We will only publish funding information present in the Funding Statement section of the online submission form. 
Please remove any funding-related text from the manuscript and let us know how you would like to update your Funding Statement. Currently, your Funding Statement reads as follows: 
    "This study was supported by the Korea Institute of Oriental Medicine [grant number KSN2121220]."**

**Please include your amended statements within your cover letter; we will change the online submission form on your behalf.**

We don’t want to change funding information. We removed the description on fuding at acknowledgment section. We added it in the online submission system.

**3. Thank you for stating the following financial disclosure: 
   "This study was supported by the Korea Institute of Oriental Medicine [grant number KSN2121220]."**
 
Please state what role the funders took in the study.  If the funders had no role, please state: "The funders had no role in study design, data collection and analysis, decision to publish, or preparation of the manuscript." 
If this statement is not correct you must amend it as needed. 
Please include this amended Role of Funder statement in your cover letter; we will change the online submission form on your behalf.

Funders of this study did noting so we added following statement in the submission system.

The funders had no role in study design, data collection and analysis, decision to publish, or preparation of the manuscript.

**4. In the online submission form, you indicated that "The data is available upon request and review by the National Health Insurance Service of Korea."**

**All PLOS journals now require all data underlying the findings described in their manuscript to be freely available to other researchers, either 1. In a public repository, 2. Within the manuscript itself, or 3. Uploaded as supplementary information.
This policy applies to all data except where public deposition would breach compliance with the protocol approved by your research ethics board. If your data cannot be made publicly available for ethical or legal reasons (e.g., public availability would compromise patient privacy), please explain your reasons on resubmission and your exemption request will be escalated for approval.**

The database data from the National Health Insurance Service undergoes a special procedure for anonymization and can only be accessed at a designated location for a limited time period. As of September 2024, the access rights to the data we requested have already been revoked, making it inaccessible. Considering that health insurance data contains sensitive personal information and access is not always permitted to protect privacy, we have described it as follows:

The data included in this study from the National Health Insurance Service in Korea can only be accessed at a designated location for a limited time period for protection of personal information so it is available upon request and review by the National Health Insurance Service of Korea.

**5. Please ensure that you refer to Figure 2 in your text as, if accepted, production will need this reference to link the reader to the figure.**

We added it in the manuscript.

**6. Please remove your figures from within your manuscript file, leaving only the individual TIFF/EPS image files, uploaded separately. These will be automatically included in the reviewers’ PDF.**

We removed them.

**7. Please include captions for your Supporting Information files at the end of your manuscript, and update any in-text citations to match accordingly. Please see our Supporting Information guidelines for more information:** [**http://journals.plos.org/plosone/s/supporting-information**](http://journals.plos.org/plosone/s/supporting-information)**.**

We added captions for Supporting information files as follows:

Supporting Information files

Supplementary Table 1. Study information

COVID, coronavirus disease-2019; NSAIDs, non-steroidal anti-inflammatory drugs; AIDS, acute immunodeficiency syndrome; HIV, human immunodeficiency virus.

Supplementary Table 2. Univariate and multivariate logistic models of medication exposure between long COVID cases and controls with duration of COVID as a covariate

Model 1, univariate; Model 2, adjusted for COVID duration; Model 3, adjusted for COVID duration, sex, and age group; Model 4, adjusted for COVID duration, sex, age group, and region; Model 5, adjusted for duration, sex, age group, region, and CCI; Model 6, adjusted for COVID duration, sex, age group, region, CCI, and underlying comorbidities

^1^Day of COVID diagnosis - 14 days after COVID diagnosis

^2^15 days since COVID diagnosis - end of follow-up (diagnosis of long COVID, diagnosis of other COVID episodes, or end of observation period

**p* <0.05; ***p* <0.01; ****p* <0.001

NSAIDs, nonsteroidal anti-inflammatory drugs; CI, confidence interval; OR, odds ratio; APAP, acetaminophen or paracetamol

Supplementary Table 3. Univariate and multivariate logistic models comparing single NSAID users with acetaminophen or paracetamol (APAP) users and combined nonsteroidal anti-inflammatory drugs + APAP with APAP users

Model 1: univariate; Model 2: adjusted for sex and age group; Model 3: adjusted for sex, age group, and region; Model 4: adjusted for sex, age group, region, and CCI; Model 5: adjusted for sex, age group, region, CCI, and underlying comorbidities

^1^Day of COVID diagnosis - 14 days after COVID diagnosis

^2^15 days since COVID diagnosis - end of follow-up (diagnosis of long COVID, diagnosis of other COVID episodes, or end of observation period

**p* <0.05; ***p* <0.01; ****p* <0.001

NSAIDs, nonsteroidal anti-inflammatory drugs; CI, confidence interval; OR, odds ratio; APAP, acetaminophen or paracetamol

**8. Please review your reference list to ensure that it is complete and correct. If you have cited papers that have been retracted, please include the rationale for doing so in the manuscript text, or remove these references and replace them with relevant current references. Any changes to the reference list should be mentioned in the rebuttal letter that accompanies your revised manuscript. If you need to cite a retracted article, indicate the article’s retracted status in the References list and also include a citation and full reference for the retraction notice.**

**There is no retracted reference. Thank you for your comments.**

**Reviewer Comments**

Association between long COVID and acute usage of nonsteroidal anti-inflammatory 2 drugs (NSAIDs) in COVID-19 patients: a nationwide cohort study from the National 3 Health Insurance Service in Korea

**Concerns**

1. **The manuscript will benefit from a grammar check.**

Thank you for your comments. We already edited our manuscript before submission through a professional English editing service. But as you commented, we checked grammar once again.

2. **Rephrase your conclusion in the abstract. Should be straightforward….eg., Both NSAIDS and APAP are robustly associated with reduced risk of Long-term COVID-19…..**

Dear reviwer,

We added this point based on your comments as follows:

**Conclusion:** While this study suggests a possible links between NSAID use in acute infection stage and higher risk of long COVID as well as both NSAIDs and acetaminophen uses during the chronic period and lower risk of long COVID, the association was not statistically significant.

3. **How and where were the controls recruited? Why did the controls have more medical conditions (covariates) than the cases? Could this have affected the outcomes observed in model 5 of Table 2? Could this potential protective effect be due to chance?**

Dear reviewer,

Thank you for your valuable comments. First of all, the control group was not radomly selected; rather, it was selected through propensity score matching from the same database and within the same time period as the observational group. These individuals had COVID-19 but did not take NSAIDs, and they were chosen in a way that controlled for other confounding factors to ensure comparibility between the two groups.

The mathing method allowed three controls per patient, matching for the exact age group, sex, CCI score, and the year and month of COVID-19 episode. For the underlying disease severity, we used the CCI score as an exact condition; this allows for a variation in the type of the underlying disease, which in our study encompassed a wide range of diseases, while matching the overall severity.

The result showed that there was no difference between the two groups except for anemia (SMD = 0.2). It is true that anemia may cause symptoms which may overlap with chronic fatigue, and the cases show higher morbidity of anemia compared to controls. However, other comorbidities which may cause more severe outcomes were balanced between the two groups, which helps us rule out the potential of confounding due to comorbidities. Furthermore, there is no direct relationship between anemia and use of painkillers, which rules out the potential unmeasured confounding between the baseline characteristics and interventions.

Based on the reviewer’s comment, we revised the manuscript as follows:

After applying PS matching to create comparable groups, a balance was achieved for demographic factors such as sex and age group, CCI, underlying comorbid conditions except for anemia, which the cases showed higher comorbidity than controls, and the year and month of the COVID-19 episode **(Table 1)**.

While there was a slight difference between cases and controls in the comorbidity of anemia (SMD = 0.20), the weak association between anemia and the use of painkillers and balance in the other diseases which may directly be associated with painkiller prescriptions, allowed us to rule out the potential of unmeasured confounding.

These individuals did not have more medical conditions, as can be confirmed from Table 1, which shows no differences between the two groups in terms of each medical condition after propensity score matching. When propensity score matching is performed, differences related to those factors between the groups disappear, which means they naturally do not affect the results. Therefore, it is difficult to consider the “potential protective effect” you mentioned as a mere coincidence

4. **Your recommendations should be more specific and not generalized. Which specific molecular or biochemical aspects are required to expand on observed relationships?**

Dear Reviewer, thank you for your comments. In fact, the exact mechanism of how long COVID develops is still not fully understood, and it is also challenging to propose a clear mechanism for how NSAIDs might worsen the symptoms of acute COVID-19 infection while having a protective effect against long COVID with prolonged use. Since this study is based on patient data, it would be difficult to specify a particular molecular or biochemical aspect for future research based solely on our results. However, to avoid any confusion in expression, we would like to clarify that section by rephrasing it as follows. We hope this meets your expectations.

Future research is crucial to further explore the effect of NSAID in the acute stage of COVID-19 infection and its molecular mechanism on the incidence of long COVID.
