# Supplementary material for: Association between long COVID and nonsteroidal anti-inflammatory drug use by patients with acute-phase COVID-19: A nationwide Korea National Health Insurance Service cohort study
Source: PLoS One. 2024 Nov 22;19(11):e0312530. doi: 10.1371/journal.pone.0312530 (PMC11584118; doi:10.1371/journal.pone.0312530)
Supplement: Attachment — Submitted filename: Rebuttal letter_2nd.docx [file pone.0312530.s007.docx]

Reply to the editorial comment

**Whiles the manuscript has improved significantly in terms of scientific content, the entire manuscript will still benefit from English-language editing. The Journal provides such services.

I use the Abstract as example; "While this study suggests a possible links between NSAID use in acute infection stage and higher risk of long COVID as well as both NSAIDs and acetaminophen uses during
the chronic period and lower risk of long COVID,......"**

Dear editor

Thank you for your comments. We edited our manuscript in a professional editing service and you can check the certificate by the company in the attached file.

We revise the abstract section as you suggested:

*Although this study suggests a possible link between NSAID use in the acute COVID-19 infection stage and a higher risk of long COVID as well as both NSAID and acetaminophen use during the chronic COVID-19 period and a lower risk of long COVID, the association was not statistically significant.*
